# Characterization of Two Highly Arsenic-Resistant Caulobacteraceae Strains of *Brevundimonas nasdae*: Discovery of a New Arsenic Resistance Determinant

**DOI:** 10.3390/ijms23105619

**Published:** 2022-05-17

**Authors:** Xiaojun Yang, Yuanping Li, Renwei Feng, Jian Chen, Hend A. Alwathnani, Weifeng Xu, Christopher Rensing

**Affiliations:** 1 College of Life Sciences, Fujian Agriculture and Forestry University, Fuzhou 350002, China; yangxiaojun0828@163.com; 2 Institute of Environmental Microbiology, College of Resources and Environment, Fujian Agriculture and Forestry University, Fuzhou 350002, China; yuanpingli@fafu.edu.cn (Y.L.); frwzym@aliyun.com (R.F.); 3 Department of Cellular Biology and Pharmacology, Herbert Wertheim College of Medicine, Florida International University, Miami, FL 33199, USA; jianchen@fiu.edu; 4 Department of Botany and Microbiology, College of Science, King Saud University, Riyadh 11451, Saudi Arabia; wathnani@ksu.edu.sa; 5 Joint International Research Laboratory of Water and Nutrient in Crop, College of Resources and Environment, Fujian Agriculture and Forestry University, Fuzhou 350002, China

**Keywords:** *Brevundimonas nasdae*, arsenic resistance, *folE*, GTP-Cyclohydrolase I, heterologous expression

## Abstract

Arsenic (As), distributed widely in the natural environment, is a toxic substance which can severely impair the normal functions in living cells. Research on the genetic determinants conferring functions in arsenic resistance and metabolism is of great importance for remediating arsenic-contaminated environments. Many organisms, including bacteria, have developed various strategies to tolerate arsenic, by either detoxifying this harmful element or utilizing it for energy generation. More and more new arsenic resistance (*ars*) determinants have been identified to be conferring resistance to diverse arsenic compounds and encoded in *ars* operons. There is a hazard in mobilizing arsenic during gold-mining activities due to gold- and arsenic-bearing minerals coexisting. In this study, we isolated 8 gold enrichment strains from the Zijin gold and copper mine (Longyan, Fujian Province, China) wastewater treatment site soil, at an altitude of 192 m. We identified two *Brevundimonas nasdae* strains, Au-Bre29 and Au-Bre30, among these eight strains, having a high minimum inhibitory concentration (MIC) for As(III). These two strains contained the same *ars* operons but displayed differences regarding secretion of extra-polymeric substances (EPS) upon arsenite (As(III)) stress. *B. nasdae* Au-Bre29 contained one extra plasmid but without harboring any additional *ars* genes compared to *B. nasdae* Au-Bre30. We optimized the growth conditions for strains Au-Bre29 and Au-Bre30. Au-Bre30 was able to tolerate both a lower pH and slightly higher concentrations of NaCl. We also identified *folE*, a folate synthesis gene, in the *ars* operon of these two strains. In most organisms, folate synthesis begins with a FolE (GTP-Cyclohydrolase I)-type enzyme, and the corresponding gene is typically designated *folE* (in bacteria) or *gch1* (in mammals). Heterologous expression of *folE*, cloned from *B. nasdae* Au-Bre30, in the arsenic-hypersensitive strain *Escherichia coli* AW3110, conferred resistance to As(III), arsenate (As(V)), trivalent roxarsone (Rox(III)), pentavalent roxarsone (Rox(V)), trivalent antimonite (Sb(III)), and pentavalent antimonate (Sb(V)), indicating that folate biosynthesis is a target of arsenite toxicity and increased production of folate confers increased resistance to oxyanions. Genes encoding Acr3 and ArsH were shown to confer resistance to As(III), Rox(III), Sb(III), and Sb(V), and ArsH also conferred resistance to As(V). Acr3 did not confer resistance to As(V) and Rox(V), while ArsH did not confer resistance to Rox(V).

## 1. Introduction

Arsenic (As) is a metalloid occurring all over the world, and ranks at the top of the US Priority List of Hazardous Substances (http://www.atsdr.cdc.gov/SPL/index.html, accessed on 15 December 2021). The concentrations of arsenic by weight in the crustal crust and sea water are around 2100 and 2.3 μg/kg, respectively (http://www.webelements.com/arsenic/geology.html, accessed on 15 December 2021). Arsenic can be accumulated and transferred via natural and anthropogenic activities, which finally enter the biosphere, in both terrestrial and oceanic environments, ending up as a toxin to organisms. It has been estimated that around 150 million people in the world are exposed to higher than 50 μg/L of As in their drinking water during daily life [1]. Exposure to arsenic leads to numerous diseases, such as diabetes, peripheral vascular disease, and even cancer [2,3].

In the environment, inorganic arsenic exists in various chemical forms, such as arsenate (As(V)), arsenite (As(III)), elemental arsenic, and arsine. Due to differences in *Eh* and pH, As(V) and As(III) are the predominant forms in the oxic and in the reducing environment, respectively [4]. As(III) is more toxic than As(V) owing to the high affinity and capability to form metallic compounds, and As(III) was present in significant amounts globally much earlier than As(V). Organic arsenic compounds also exist in various forms, such as pentavalent roxarsone (Rox(V)), methylarsenate (MAs(V)), and the trivalent roxarsone (Rox(III)) and methylarsenite (MAs(III)) [5]. Antimony (Sb) and As are group VA elements on the periodic table, and Sb shares some similar chemical and toxicological properties with As, which have been extensively studied [6]. Sb exists in four oxidation states (+V, +III, 0, and −III), of which pentavalent antimonate (Sb(V)) and trivalent antimonite (Sb(III)) are the prevalent forms in the environment [7]. Microbial communities have been shown to play an important role in the arsenic and antimony global biocycles [8,9]. As(III) efflux and methylation are responsible for the majority of As(III) tolerance and detoxification pathways in most organisms [10,11]. More and more new arsenic resistance (*ars*) genes have been found to confer resistance to arsenic and various organic arsenic compounds and are encoded in *ars* operons [12,13,14,15,16,17,18]. Acr3 and ArsB are the most widespread *ars* operon-encoded gene products, which were shown to pump As(III) from the cytosol across the cytoplasmic membrane into the periplasm or extracellular medium [10]. ArsG, ArsJ, ArsK, ArsP, ArsQ and ArsW have been shown to be efflux transporters encoded on *ars* operons in various organisms, with each mediating transport of different types of organic arsenicals [12,13,14,19,20,21]. ArsC, an As(V) reductase, has been shown to quickly reduce intracellular As(V) to As(III) [22]. ArsE, ArsF, ArsM, ArsH, ArsI, ArsN, ArsO, ArsU, ArsV and ArsL were shown to confer resistance to organic arsenical [12,13,14,15,16,17,18,23,24].

In this study, *Brevundimonas nasdae* strains Au-Bre29 and Au-Bre30 were isolated from Zijin gold- and copper-mining soil, with high multiple heavy-metal(loid)s resistance, but behaving differently regarding secretion of extra-polymeric substances (EPS) upon As(III) stress. We further characterized different genes conferring resistance to various arsenic and antimony species and compounds.

## 2. Results

### 2.1. The Soil Sample from the Zijin Gold and Copper Mine Contained Heavy Metals and Metalloids at Very High Concentrations

The heavy-metal(loid)s concentrations in the sampling soil of the Zijin gold and copper mine were determined, respectively (Table 1). The concentrations of As, Cd, Cu and Pb in the sampling site were significantly higher than the third level of the environmental quality standard for soils of China (GB 15618-1995).

### 2.2. Eight Highly Arsenite-Resistant Strains Were Isolated and Identified

Eight strains, named Au-Aci5, Au-Bre4, Au-Bre29, Au-Bre30, Au-Lys6, Au-Mic3, Au-Pse14 and Au-Pse15, respectively, were isolated from the Au(III) enrichment on R2A plates containing 160 μM of Au(III). Three of the eight isolates were shown to belong to the genus of *Brevundimonas* after blasting the 16S ribosomal RNA (rRNA) sequence to the database of the National Center for Biotechnology Information (NCBI). Au-Aci5 showed 100% similarity to *Acinetobacter seifertii* CMCC(B)25090, Au-Bre4 had 99.92% similarity to *Brevundimonas vesicularis* DCB19, Au-Bre29 and Au-Bre30 had 99.85% and 99.78% similarity to *Brevundimonas nasdae* DSS_C4, Au-Lys6 had 99.93% similarity to *Lysinibacillus fusiformis* LSP22, Au-Mci3 showed 100% similarity to *Microbacterium binotii* CIP 102116, and Au-Pse14 and Au-Pse15 had 99.93% similarity to *Pseudomonas frederiksbergensis* JS1015, respectively. The 16S rRNA sequences of the eight isolates were deposited to the NCBI database under accession numbers: OK178958, OK178960, OK182900, OK182930, OK178961, OK178958, OK271131 and OK560628, respectively (Table 2). Based on the 16S rRNA alignment results and the phenotype of the eight isolates, strains Au-Bre29 and Au-Bre30 with high similarity to *Brevundimonas nasdae* were selected for the following study.

### 2.3. High Arsenic Resistance and Heavy-Metal Resistance Were Determined in B. nasdae Strains Au-Bre29 and Au-Bre30

After incubation on R2A plates containing various concentrations of Au(III), Cu(II), Zn(II), Cd(II) and As(III) at 28 °C for 3 days, the minimum inhibitory concentration (MIC) of strains Au-Bre29 and Au-Bre30 to heavy metal(loid)s was determined. Different isolates displayed differing abilities to resist different metal(loid)s, but all the strains conferred a high resistance to Au(III) (>100 μM) (Table 3). Furthermore, strain Au-Bre29 was found to be more resistant to Cu(II) and less resistant to As(III) compared to strain Au-Bre30. The EPS production of strain Au-Bre30 on R2A plates containing As(III) was significantly higher than in strain Au-Bre29, indicating that strains Au-Bre30 and Au-Bre29 might have a partially different response to As(III) exposure.

### 2.4. The Growth Conditions for B. nasdae Strains Au-Bre29 and Au-Bre30 could Be Optimized

**Figure 1:**
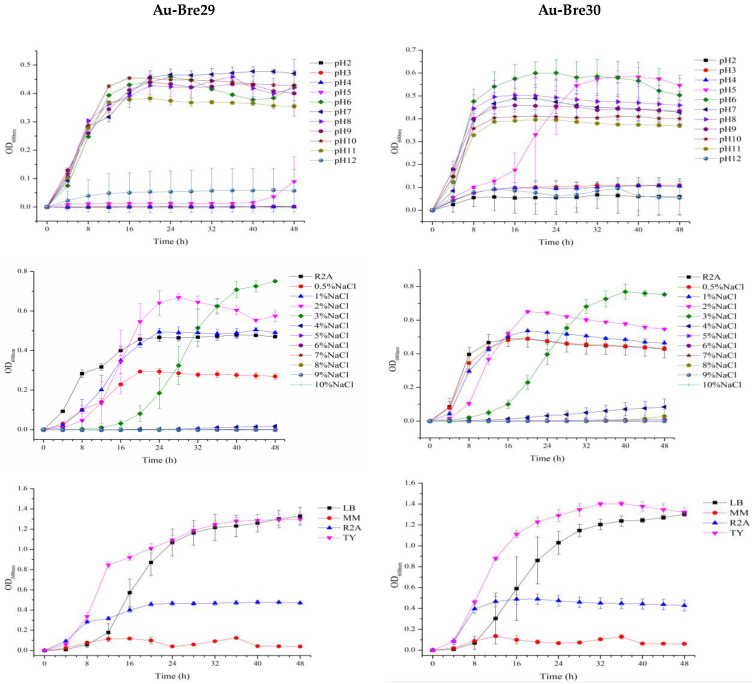
Optimized growth patterns of *B. nasdae* strains Au-Bre29 and Au-Bre30. Au-Bre29 and Au-Bre30 were cultured individually in R2A medium with different pH levels (2–12, additional NaCl = 0%), different additional NaCl concentrations (0–10%, pH = 7.0), and different media (MM, R2A, TY and LB, pH = 7.0). Performed in the Bioscreen C microbiology reader at 28 °C for 48 h, and the optical densities at 600nm (OD_600nm_) were automatically measured at 4 h intervals. The growth curves display the results of three replicates, and error bars represent the standard error of the average values.

The growth of bacterial strains depends on growth parameters including the behavior of strains Au-Bre29 and Au-Bre30, and a set of experiments were conducted. As shown in Figure 1, the best medium for growing strains Au-Bre29 and Au-Bre30 is TY medium. Strain Au-Bre29 was able to grow in R2A medium with pH ranging from 6 to 11, and strain Au-Bre30 was able to tolerate a pH ranging from 5 to 11. Both strains were able to grow in R2A medium with additional NaCl concentrations ranging from 0 to 3%. All these results indicated that there was little influence of these parameters on the growth patterns of both *B. nasdae* strains, Au-Bre29 and Au-Bre30.

### 2.5. Genome Analysis of B. nasdae Strains Au-Bre29 and Au-Bre30

The complete genome was sequenced to be able to compare *B. nasdae* strains Au-Bre29 and Au-Bre30 with a focus on heavy-metal resistance, especially As(III) resistance determinants. The circular maps of the complete genome of both Au-Bre29 and Au-Bre30 are shown in Figure S1. Compared to strain Au-Bre30, strain Au-Bre29 contained one extra plasmid. Genome size (Kb), genes existing on the forward strand and reverse strand, repetitive sequence, annotated tRNA (blue) and rRNA (purple), GC content, and GC skew are also presented in Figure S1. As show in Table S1, the G+C content of Au-Bre29 and Au-Bre30 is 65.69% and 65.8%, respectively. Strain Au-Bre29 was shown to have 3665 coding sequence (CDS) and 53 tRNAs, while strain Au-Bre30 was shown to have 3577 CDS and 53 tRNAs. A total of 3731 genes were found on the genome of strain Au-Bre29, and 3618 genes were assigned with function prediction, 1772 genes were predicted to encode functions involved in the Kyoto Encyclopedia of Genes and Genomes (KEGG) pathways, and 531 genes were assigned to signal peptides. As in strain Au-Bre29, strain Au-Bre30 was shown to contain 3643 genes, with 3531, 3474, 1751 and 523 genes associated with protein coding sequence, function prediction, KEGG pathway, and signal peptides, respectively. Furthermore, the presence of Clustered Regularly Interspaced Short Palindromic Repeats (CRISPR) loci has been shown to increase the genomic stability of strains, allowing them to adapt to various environments [25].

As shown in Figure S2, there are many hypothetical proteins encoded on the plasmid of Au-Bre29. Other genes encoded conjugative transfer system proteins, such as TraC, TraE, TraF, TraG and TraH. There was no arsenic resistance gene on the plasmid of Au-Bre29. As Figure S3 shows, the Mauve alignment showed high synteny and similarity among the genomes of *B. nasdae* strains Au-Bre29 and Au-Bre30.

### 2.6. ars Operons in B. nasdae Strains Au-Bre29 and Au-Bre30 Are Highly Similar

**Figure 2:**
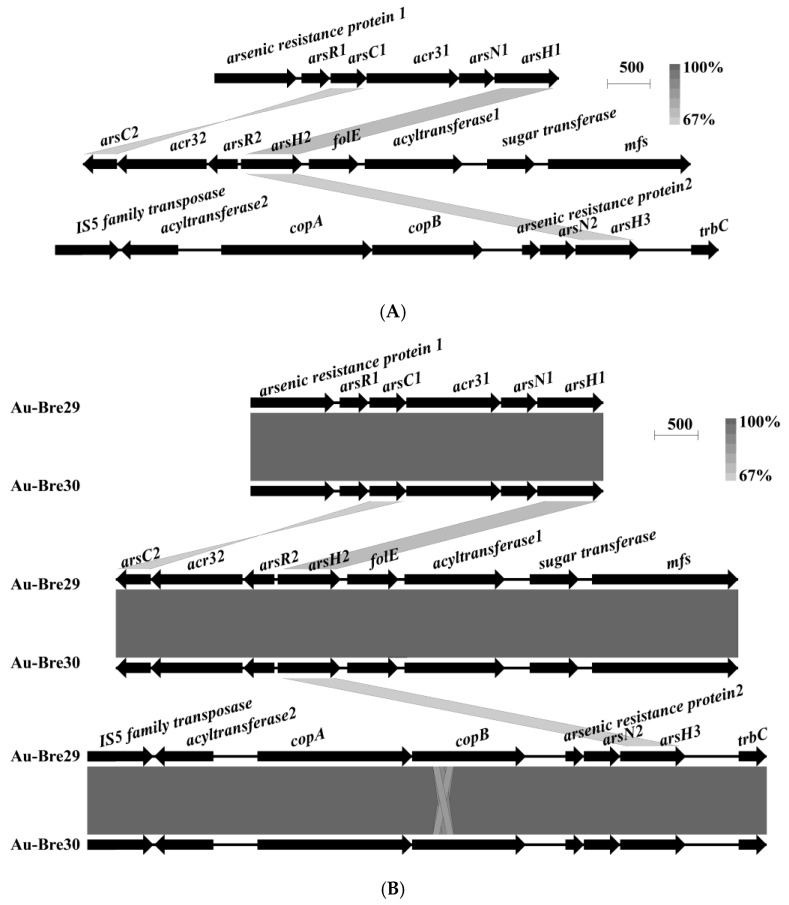
*ars* operons in *B. nasdae* strain Au-Bre30 (**A**), and the alignment between the *ars* operons present on the chromosome of *B. nasdae* strains Au-Bre29 and Au-Bre30 (**B**). Physical maps were generated by using Easyfig.

Three ars operons were identified on each of the chromosomes of strains Au-Bre29 and Au-Bre30: *arsenic resistance protein*1–*arsR*1–*arsC*1–*acr3*1–*arsN*1–*arsH*1 (*ars*1), *arsC*2–*acr3*2–*arsR*2–*arsH*2–*folE*–*acyltransferase*1–*sugar transferase*–*mfs* (*ars*2), and *IS5 family transposase*–*acyltransferase*2–*copA*–*copB*–*arsenic resistance protein*2–*arsN*2–*arsH*3–*trbC* (*ars*3) (Figure 2A). All of the *ars* operons were only found on the chromosome and not on the plasmid. Interestingly, a gene annotated as *folE* was located near *arsH* in the *ars* operon. Interestingly, the expression of *folE* was upregulated in strain *Paenibacillus taichungensis* NC1 exposed to 0.2 and 2 mM of As(III), indicating that FolE is predicted to have a role in conferring arsenic resistance in both strains [26]. After comparison of the genome sequences of the two strains, no major difference was found expect for the presence of the *copA* and *copB* genes adjacent to one of the *ars* operons (Figure 2B).

### 2.7. Heterologous Expression of folE Confers Resistance to As(III), As(V), Rox(III), Rox(V), Sb(III), and Sb(V)

**Figure 3:**
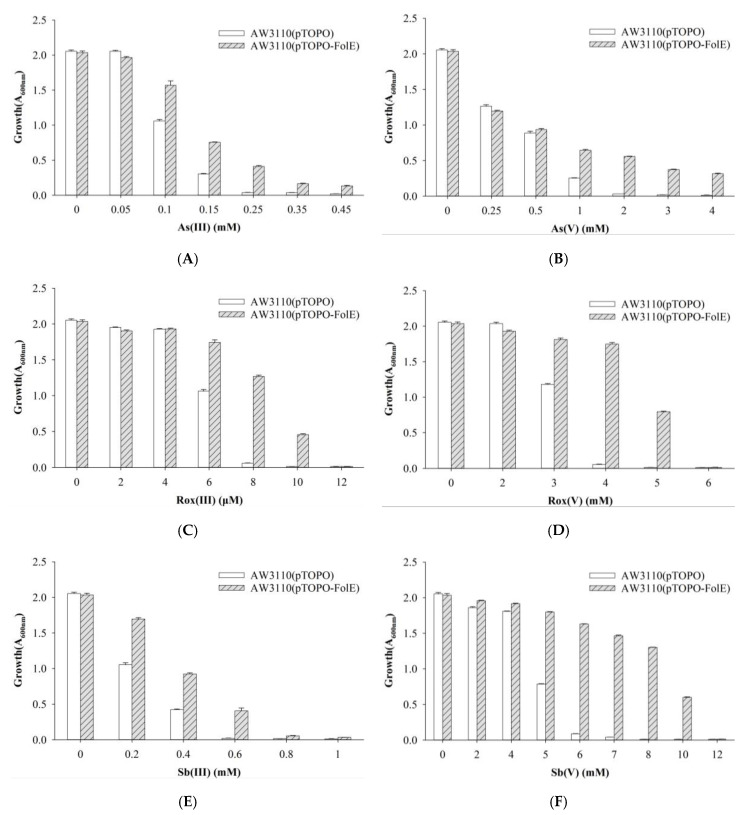
Growth of *E. coli* AW3110 bearing pTOPO and pTOPO-FolE with the indicated concentrations of As(III) (**A**), As(V) (**B**), Rox(III) (**C**), Rox(V) (**D**), Sb(III) (**E**), and Sb(V) (**F**). The A_600__nm_ was measured after 24 h of growth at 37 °C and 220 rpm. The graphs show the results of three replicates, and error bars represent the standard error of the average values.

*E. coli* AW3110, the arsenic-hypersensitive strain with an *arsRBC* deletion, contained the control pTOPO plasmid or the pTOPO plasmid carrying *folE* to determine the function of FolE. *E. coli* AW3110 carrying plasmid pTOPO was able to grow at concentrations of As(III), Rox(III) and Sb(III) up to 0.15 mM, 6 μM, and 0.4 mM, respectively. *E. coli* AW3110 bearing plasmid pTOPO-FolE was able to grow when exposed to concentrations higher than 0.45 mM, 10 μM, and 0.6 mM of As(III), Rox(III), and Sb(III), respectively. *E. coli* AW3110 carrying plasmid pTOPO was able to tolerate As(V), Rox(V), and Sb(V) up to 1, 3, and 5 mM, respectively. *E. coli* AW3110 bearing plasmid pTOPO-FolE was able to grow when exposed to concentrations higher than 4, 5 and 10 mM of As(V), Rox(V) and Sb(V), respectively. Cells expressing *folE* were not only more resistant to organic and inorganic arsenic but also to trivalent and pentavalent antimony (Figure 3).

**Figure 4:**
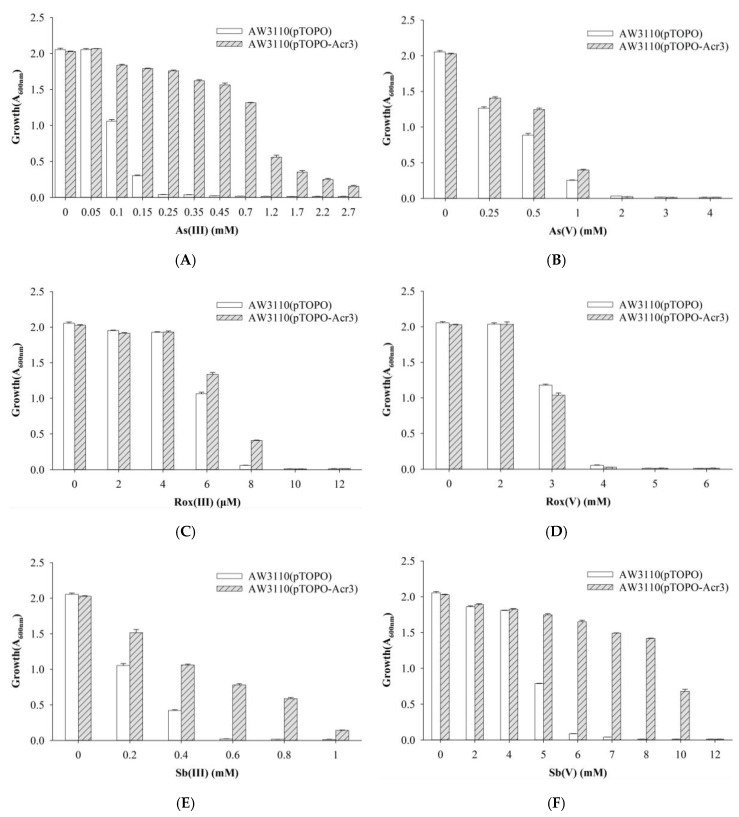
Growth of *E. coli* AW3110 bearing pTOPO and pTOPO-Acr3 with the indicated concentrations of As(III) (**A**), As(V) (**B**), Rox(III) (**C**), Rox(V) (**D**), Sb(III) (**E**), and Sb(V) (**F**). The A_600__nm_ was measured after 24 h of growth at 37 °C and 220 rpm. The graphs show the results of three replicates, and error bars represent the standard error of the average values.

**Figure 5:**
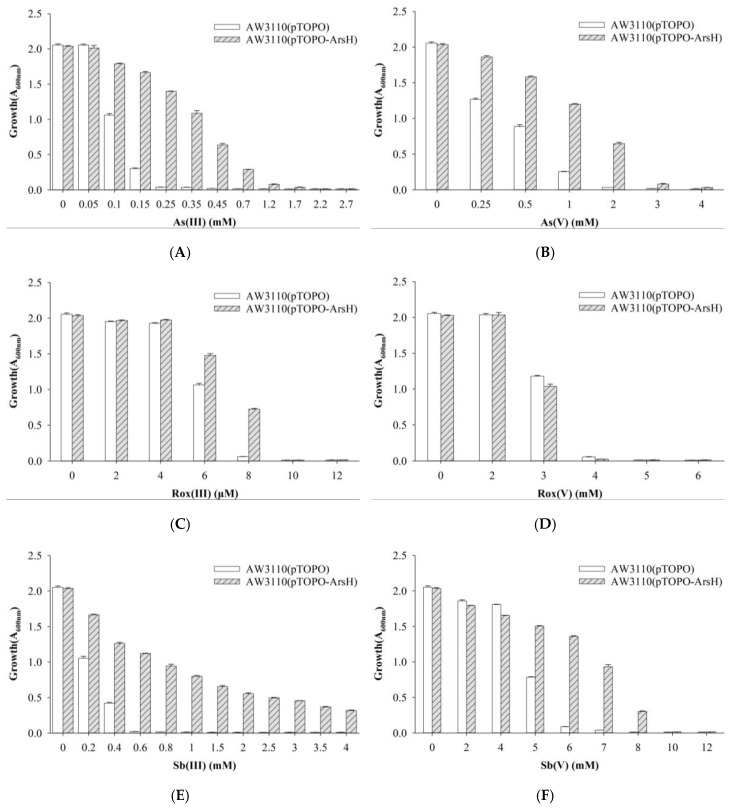
Growth of *E. coli* AW3110 bearing pTOPO and pTOPO-ArsH with the indicated concentrations of As(III) (**A**), As(V) (**B**), Rox(III) (**C**), Rox(V) (**D**), Sb(III) (**E**), and Sb(V) (**F**). The A_600__nm_ was measured after 24 h of growth at 37 °C and 220 rpm. The graphs show the results of three replicates, and error bars represent the standard error of the average values.

To investigate the function of the *acr3-* and *arsH*-encoded gene products also present in the *ars*2 operon, *acr3* and *arsH* from strain Au-Bre30 were cloned into the pTOPO plasmid. The arsenic-sensitive strain *E. coli* AW3110 carrying plasmid pTOPO-Acr3 was able to grow in 1.2 mM, 8 μM, 0.8 mM, and 10 mM of As(III), Rox(III), Sb(III), and Sb(V), respectively, while the strain containing the empty pTOPO did not grow under the same conditions. However, *E. coli* AW3110 carrying plasmid pTOPO-Acr3 or pTOPO did not display significant differences when exposed to As(V) and Rox(V). Expression of *acr3* conferred very significant resistance to As(III), Sb(III), and Sb(V), conferred a small increase in resistance to Rox(III), but did not confer resistance to As(V) and Rox(V) (Figure 4). *E. coli* AW3110 carrying plasmid pTOPO-ArsH was able to grow in 0.45 mM, 8 μM, 3 mM, 2 mM and 7 mM of As(III), Rox(III), Sb(III), As(V) and Sb(V), respectively, while *E. coli* AW3110 containing pTOPO did not grow under the same conditions. However, *E. coli* AW3110 carrying plasmid pTOPO-ArsH and pTOPO showed no difference when exposed to As(V). Furthermore, we were able to show that expression of *arsH* conferred very significant resistance to As(III) and Sb(III), conferred significant resistance to Rox(III), As(V) and Sb(V), but did not confer resistance to Rox(V) (Figure 5).

## 3. Discussion

Mine soil with extremely high concentrations of multiple heavy metals has been treated as one of the hot spots to study how organisms adapt and survive under these hazardous conditions. The Zijin gold and copper mine is the biggest gold mine in China, with a long history of mining indicating high levels of long-term heavy-metal contamination. In the present study, eight strains were isolated via Au-enrichment isolation, with three of the eight strains belonging to the genus *Brevundimonas*, with two of the three strains displaying high similarity to *Brevundimonas nasdae* but having a distinct phenotype on R2A plates, such as different levels of EPS secretion. This indicated the presence of genomic differences between *B. nasdae* strains Au-Bre29 and Au-Bre30 in their adaptation to heavy-metal-contaminated environments. Complete genome sequencing indicated that *B. nasdae* strains Au-Bre29 and Au-Bre30 both contain 3 *ars* operons conferring high arsenic resistance. These three *ars* operons, *ars*1, *ars*2 and *ars*3, with the *ars*3 operon in the vicinity of *copA* and *copB*, are located on conjugative transposons, and the *ars*2 operon contains a gene named *folE* adjacent to other genes with putative arsenic-related functions. Furthermore, these two strains show no difference in *ars* operons, as only one extra plasmid without any *ars* genes was found in *B. nasdae* Au-Bre29. Cloning of *folE* into an expression vector for heterologous expression in arsenic-hypersensitive strain AW3110 conferred resistance to As(III), As(V), Rox(III), Rox(V), Sb(III), and Sb(V). These results demonstrate that *folE*, encoding GTP-Cyclohydrolase I, is a novel arsenic resistance gene, that confers resistance to both trivalent and prevalent organic and inorganic arsenic.

Arsenic and antimony from both natural and anthropogenic sources contaminate the environment. As an element, arsenic and antimony cannot be destroyed and will always be present in the environment, but bio-transformations have been able to modify their physical location and chemical speciation. In T24 cells, which were derived from a human urinary bladder carcinoma lacking the ability of As(III) methylation, the relative toxicities were determined to be MAs(III) > DMAs(III) ≈ As(III) >> AsV > DMAs(V) > MAs(V) > Rox(V) [27]. The general order of toxicity for Sb species is presented as: Sb(III) > Sb(V) > organoantimonials [28]. MAs(III) and Rox(III) are likely oxidized by ArsH, resulting in reduced toxicity in *E. coli* AW3110 expressing *arsH* (Figure 5).

The *arsH* gene had previously been cloned from a rhizosphere bacterium, and its gene product, ArsH, was shown to be an NADPH-FMN-dependent oxidoreductase that oxidizes highly toxic MAs(III) to relatively nontoxic MAs(V) [18]. ArsH relieved the toxicity of As species by mediating the reduction of ROS produced in vivo upon exposure to the oxyanion, e.g., by generating FMNH_2_ to fuel ROS-quenching activities [29]. A recent study found that ArsH was also significantly upregulated in the oxidation process of Sb(III) [30], suggesting the possibility of its participation in the oxidation of Sb(III) in *Klebsiella aerogenes* X [31]. *Pseudomonas putida arsH* expressed in *E. coli* conferred resistance to MAs(III), Rox(III), and phenylarsenite (PhAs(III)) [25]. Our results show that *arsH* not only conferred resistance to Sb(III) and Rox(III), but also As(III), As(V) and Sb(V), strongly suggesting it not only oxidizes MAs(III) and Rox(III) to MAs(V) and Rox(V), respectively, but also protects against ROS (Figure 5).

GTP-Cyclohydrolase I (GTPCHI, EC 3.5.4.16) belongs to the cyclohydrolase family of enzymes, ranging from 190 to 250 amino acid residues, and is invariably present in many biological systems, including bacteria, fungi, plants, and higher animals, and shown to be conserved on eukaryotic as well as on bacterial genomes [32]. GTPCHI is the rate-limiting enzyme involved in the biosynthesis of tetrahydrobiopterin (BH4), a key cofactor necessary for nitric oxide synthase and for the hydroxylases that are involved in the production of catecholamines and serotonin [33]. GTPCHI requires zinc ions as a cofactor and involves two hydrolyses and an Amadori rearrangement, yielding formic acid as a byproduct [34]. GTPCHI is encoded by the *folE* gene. Folates are essential cofactors for one-carbon transfer reactions in most living organisms [35]. There is mounting evidence that folate influences arsenic metabolism [36], increasing methylation of inorganic to DMAs [37]. Overexpression of *folE* ensures a sufficient supply of folate. The expression of GTPCHI from *E. coli* in transgenic *Arabidopsis* resulted in a 1250-fold and 2- to 4-fold enhancement of pterins and folates, respectively [38]. Overexpression of the *folKE* gene in *Lactococcus lactis* increased folate production by almost 3-fold [39]. The presence of folate is essential for life and the folate biosynthesis pathway appears to be a main target of toxicity of various arsenical species. We therefore propose that overexpression of *folE* protects cells by ensuring that sufficient folate is produced even under exposure to various arsenic and antimony species (Figure 3).

Acr3 is an As(III) and Sb(III) efflux pump, which is a member of the BART (bile/arsenite/riboflavin transporter) superfamily and includes members found in bacteria, archaea, and fungi, and is more widely distributed than members of the ArsB family [40,41]. The Acr3 protein is present in the *Saccharomyces cerevisiae* plasma membrane and pumps As(III), but not As(V), Sb(III), tellurite (Te(III)), Cd(II), or phenylarsine oxide out of the cell in response to the proton motive force [42]. Cells of *E. coli* expressing AmAcr3-1 or CgAcr3-1 confer As(III) resistance. In addition, everted membrane vesicles prepared from *Corynebacterium glutamicum* expressing Acr3-1 were shown to transport As(III) but not Sb(III) using NADH and an electron donor for the respiratory chain [41,43]. Our results show that Acr3 not only conferred resistance to As(III), but also to Sb(III) (Figure 4). Resistance to high levels of Sb(V) might be due to low levels of reduction of Sb(V) to Sb(III) and subsequent efflux of Sb(III).

## 4. Materials and Methods

### 4.1. Strain Isolation

One gram of the soil from the Zijin gold and copper mine (Longyan, Fujian Province, China) wastewater treatment (25°09.724′ N, 116°23.258′ E), at an altitude of 192 m, was added into a 50 mL flask containing 9 mL of phosphate-buffered saline solution (pH 7.0, 0.2 M). The flask was incubated with shaking at 28 °C, 180 rpm for 6 h, to activate the microbial life inside the soil. After 6 h, 100 μL of the above soil solution was plated on R2A plates containing 10 μM of Au(III), and incubated at 28 °C until colonies appeared. Then, single colonies, with different phenotypes, were individually picked up and transferred to an R2A plate containing increasingly higher concentrations of Au(III). The Au(III) concentration ranged from 10 to 160 μM on the R2A plates. The strain grown on the R2A plate containing 160 μM of Au(III) was kept and purified by plating on the R2A plate with the same concentration of Au(III) 3–5 times. Finally, 8 strains were harvested and stored at −80 °C in 15% glycol (V:V). MIC results showed that both strains Au-Bre29 and Au-Bre30 had high heavy-metal resistance to Au(III), Cu(II) and As(III), but with differences in As(III) and Cu(II) resistance; furthermore, strains Au-Bre29 and Au-Bre30 belong to the species *Brevundimonas nasdae*, so these two strains were selected for further study.

### 4.2. Heavy-Metal Concentration Measurements of the Sampled Soil

The soil samples were digested by a microwave digestion system according to USEPA Method 3051A (USEPA, 2007) [44], and further determined by inductively coupled plasma mass spectrometry (ICP-MS) (PerkinElmer NexION 300X, Waltham, MA, USA). 

### 4.3. Strain Characterization

#### 4.3.1. Determination of Minimum Inhibitory Concentration (MIC) of Metal(loid)s on R2A Plates

R2A plates were used for the MIC test for isolates of different metal(loid)s since not all isolates can grow in MM medium, and the TY medium contains too many nutrients. First, isolates were activated from −80 °C on the R2A plate, at 28 °C, until a single colony appeared. Then, single colonies were individually transferred into R2A medium cultured at 28 °C, at 180 rpm overnight. The OD_600nm_ of the above strain culture was adjusted to be 0.1, then diluted with R2A medium 100 times. Finally, the diluted culture was streaked on R2A plates containing 0.25–4 mM of Cu(II), 0.25–6 mM of Cd(II), and 0.25–6 mM of As(III), with a 0.25 increment, respectively. The plates were inoculated at 28 °C for 3 days. The R2A plate containing the lowest concentration of metal(loid)s, where no visible growth of strains was observed after 3 days, was recorded as the MIC.

#### 4.3.2. Molecular Identification of Isolates

For molecular identification, the genomic DNA of strain Au-Bre30 was extracted using the TIANamp Bacteria DNA kit under the guidance of the manufacturer’s instructions (Tiangen Biotech, Beijing, China). The 16S rRNA gene was amplified using the primers (27F and 1492R) with genomic DNA as a template [45]. One percent agarose gel was used to verify the amplification of the 16S rRNA product by running gel, and after that, the confirmed PCR product was sent out to the Sangon Biotech company (Shanghai, China) for sequencing. Partial 16S rRNA gene sequences were uploaded and subjected to NCBI to identify the most related species based on the most homology. A phylogenetic tree was constructed using assembled sequences of 16S rRNA genes and the neighbor-joining algorithms via MEGA X [46].

#### 4.3.3. Determination of Optimal Growth Conditions of *B. nasdae* Au-Bre29 and Au-Bre30

Single colonies of strains Au-Bre29 and Au-Bre30 that appeared on the R2A plate were transferred into liquid R2A medium and incubated with shaking at 28 °C. Thereafter, the strain culture was transferred into fresh R2A medium and cultured under the same conditions overnight. The OD_600nm_ of the above strain culture was adjusted to be 1.0 for further study. One percent of strain culture with OD_600nm_ adjusted was transferred into new R2A medium with different pH levels (2–12, additional NaCl = 0%), different additional NaCl (0–10%, pH = 7.0) concentrations, and different medium (LB, MM, R2A, and TY, pH = 7.0), individually. LB medium contained 10 g/L of tryptone, 5 g/L of yeast extract, and 10 g/L of NaCl, with pH adjusted to 7.0 by HCl or NaOH [47]. MM medium contained 2.0 g/L of sodium gluconate, 6.06 g/L of trimethylaminomethane, 4.68 g/L of NaCl, 1.49 g/L of KCl, 1.07 g/L of NH_4_Cl, 0.43 g/L of Na_2_SO_4_, 0.005 g/L of ferric ammonium citrate, 0.2 g/L of MaCl_2_·6H_2_O, 0.03 g/L of CaCl_2_·2H_2_O, 0.23 g/L of Na_2_HPO_4_·12H_2_O, 1.5 mg/L of FeCl_2_·4H_2_O, 0.19 mg/L of CoCl_2_·6H_2_O, 0.1 mg/L of MnCl_2_·4H_2_O, 62 μg/L of H_3_BO_3_, 70 μg/L of ZnCl_2_, 36 μg/L of Na_2_MoO_4_·2H_2_O, 24 μg/L of NiCl_2_·6H_2_O, and 17 μg/L of CuCl_2_·2H_2_O, with pH adjusted to 7.0 by HCl or NaOH [26]. R2A medium contained 0.5/L g of tryptone, 0.5 g/L of yeast extract, 0.5 g/L of casamino acid, 0.5 g/L of glucose, 0.5 g/L of soluble starch, 0.3 g/L of K_2_HPO_4_, 0.024 g/L of MgSO_4_, and 0.3 g/L of sodium pyruvate, with pH adjusted to 7.0 by HCl or NaOH [48]. TY medium contained 5 g/L of tryptone, 3 g/L of yeast extract, and 1.3 g/L of CaCl_2_·6H_2_O, with pH adjusted to 7.0 by HCl or NaOH [49]. Then, 200 μL of each diluted culture was loaded into separate wells of a 100-well honeycomb plate in 3 duplicates. A cover was used on the plate to prevent evaporation. Growth experiments were performed in the Bioscreen C microbiology reader (Oy Growthcurves Ab Ltd., Helsinki, Finland) at 28 °C for 48 h, and the optical densities at 600nm (OD_600nm_) were automatically measured at 4 h intervals.

#### 4.3.4. Nanopore Sequencing, Annotation, and Assembly

High-quality genomic DNA with OD260/280 values higher than 1.80 from strains Au-Bre29 and Au-Bre30 were extracted following the instructions of the TIANamp Bacteria DNA Kit (Tiangen Biotech, Beijing, China). DNA fragments with size > 20 Kb were collected and sent to Biomarker Biotech Co., Ltd. (Beijing, China) for sequencing by using the PromethION platform (R9.4.1, FLO-PRO002; Oxford Nanopore Technologies (ONT), Oxford, UK). The SQK-LSK109 kit was used to construct the DNA library under the guidance of the standard protocol of ONT. The NEBNext^®^ Ultra™ DNA Library Prep Kit (New England Biolabs, Beijing, China) was used for DNA sequencing via the Illumina Novaseq 6000 platform, with an insert size of 300 bp (paired end). The sequence was assembled by using Canu (v1.5), and annotated by the NCBI Prokaryotic Genome Annotation Pipeline (PGAP) with GenBank accession numbers CP080034-CP080035 (Au-Bre29) and CP080036 (Au-Bre30) [50].

### 4.4. Identification of Putative ars Genes

#### 4.4.1. Plasmids, Bacterial Strains, and Growth Conditions

Competent cell of *E. coli* DH5α, purchased from TransGen Biotech (Beijing, China), was used for plasmid DNA replication. The As(III)-hypersensitive strain, *E. coli* AW3110 (Δ*arsRBC::cam*) [51], was used for the following complementation studies. The PCR products of putative arsenic resistance-related genes were cloned into plasmid pTOPO by using the Zero Background pTOPO-Blunt Simple Cloning Kit under the guidance of the manufacturer (Aidlab, Beijing, China). The strains bearing the indicated plasmid were grown aerobically in LB medium containing 100 μg/mL of ampicillin (Amp). The absorbance at OD_600nm_ was used to measure the growth of the strains. The three putative arsenic resistance-related genes were designated as *folE*, *acr3*, and *arsH* from *B. nasdae* Au-Bre30. The FolE, Acr3, and ArsH had GenBank accession numbers QYC15516, QYC15513, and QYC15515, respectively, and were cloned into the pTOPO plasmid. Furthermore, the pTOPO plasmid bearing indicated genes was first transferred into DH5α to replicate, and then transferred into AW3110 to complement.

#### 4.4.2. Metal(loid)s Resistance Analysis

A single colony of *E. coli* strain AW3110 harboring the indicated plasmid was grown in LB medium containing 100 μg/mL of Amp at 37 °C, 220 rpm. After 12 h of incubation, the OD_600nm_ of strain culture was adjusted to 0.1 with LB added, and then the adjusted strain culture was transferred into new LB medium supplied with 100 μg/mL of Amp at 1% and cultured under the same conditions for around 12 h. The absorbance at OD_600nm_ of the overnight cultured strain was adjusted to 1.0. The adjusted strain culture was diluted 100-fold in LB medium containing 100 μg/mL of Amp and different concentrations of metal(loid)s and shaken for 24 h. The growth of strain *E. coli* AW3110 bearing different plasmids was measured by the value of OD_600nm_.

## 5. Conclusions

We characterized two highly arsenite-resistant *B. nasdae* strains, Au-Bre29 and Au-Bre30, and identified the novel arsenic resistance determinant *folE* in *B. nasdae* Au-Bre30, encoding GTP-Cyclohydrolase I (FolE). Heterologous expression of *folE* in the arsenic-hypersensitive *E. coli* strain AW3110 resulted in increased resistance to As(III), As(V), Rox(III), Rox(V), Sb(III), and Sb(V). Heterologous expression of *arsH* and *acr3* in AW3110 resulted in increased resistance to As(III), Rox(III), Sb(III), and Sb(V). *arsH* also conferred resistance to As(V). These results indicated that expression of *arsH* conferred resistance to ROS generated due to As or Sb exposure.

## Figures and Tables

**Table 1:** Heavy-metal concentrations in the sampling soil. Data are presented as means ± standard deviations (*n* = 3).

	Ag	As	Cd	Cr	Cu	Mn	Ni	Pb	Sb
Concentration (mg/kg)	12.86 ± 1.27	66.99 ± 18.97	89.00 ± 26.58	62.64 ± 14.24	1239.53 ± 273.32	788.96 ± 150.56	97.98 ± 18.73	526.45 ± 102.88	4.63 ± 0.24

**Table 2:** Eight Au enrichment isolates from the Zijin gold and copper mine.

Strain	Closest Relative	Similarity	GenBank Accession No.
Au-Aci5	*Acinetobacter seifertii*	100%	OK178959
Au-Bre4	*Brevundimonas vesicularis*	99.92%	OK178960
Au-Bre29	*Brevundimonas nasdae*	99.85%	OK182900
Au-Bre30	*Brevundimonas nasdae*	99.78%	OK182930
Au-Lys6	*Lysinibacillus fusiformis*	99.93%	OK178961
Au-Mic3	*Microbacterium binotii*	100%	OK178958
Au-Pse14	*Pseudomonas frederiksbergensis*	99.93%	OK271131
Au-Pse15	*Pseudomonas frederiksbergensis*	99.93%	OK560628

**Table 3:** MICs of strains Au-Bre29 and Au-Bre30 for selected heavy metal(loid)s.

Strain	Au(III) (μM)	Cu(II) (mM)	Zn(II) (mM)	Cd(II) (mM)	As(III) (mM)
Au-Bre29	600	1.25	8	0.75	5
Au-Bre30	600	1	8	0.75	6.5

## Supplementary Materials

The following supporting information can be downloaded at: https://www.mdpi.com/article/10.3390/ijms23105619/s1.

## References

[1] Brammer H., Ravenscroft P. (2009). Arsenic in groundwater: A threat to sustainable agriculture in South and South-east Asia. Environ. Int..

[2] Tseng C.H., Tseng C.P., Chiou H.Y., Hsueh Y.M., Chong C.K., Chen C.-J. (2002). Epidemiologic evidence of diabetogenic effect of arsenic. Toxicol. Lett..

[3] Chen C.J., Chen C., Wu M., Kuo T. (1992). Cancer potential in liver, lung, bladder and kidney due to ingested inorganic arsenic in drinking water. Br. J. Cancer.

[4] Zhu Y.G., Yoshinaga M., Zhao F.J., Rosen B.P. (2014). Earth Abides Arsenic Biotransformations. Annu. Rev. Earth Planet. Sci..

[5] Stolz J.F., Basu P., Santini J.M., Oremland R.S. (2006). Arsenic and selenium in microbial metabolism. Annu. Rev. Microbiol..

[6] Nies D.H. (1999). Microbial heavy-metal resistance. Appl. Microbiol. Biotechnol..

[7] Chen B. (2002). Antimony in the environment: A review focused on natural waters: I. Occurrence. Earth Sci. Rev..

[8] Chen J., Rosen B.P. (2020). The Arsenic Methylation Cycle: How Microbial Communities Adapted Methylarsenicals for Use as Weapons in the Continuing War for Dominance. Front. Environ. Sci..

[9] Li J., Wang Q., Oremland R.S., Kulp T.R., Rensing C., Wang G. (2016). Microbial Antimony Biogeochemistry: Enzymes, Regulation, and Related Metabolic Pathways. Appl. Environ. Microbiol..

[10] Yang H.C., Fu H.L., Lin Y.F., Rosen B.P. (2012). Pathways of arsenic uptake and efflux. Curr. Top. Membr..

[11] Giovannoni S.J., Halsey K.H., Saw J., Muslin O., Suffridge C.P., Sun J., Lee C.P., Moore E.R., Temperton B., Noell S.E. (2019). A Parasitic Arsenic Cycle That Shuttles Energy from Phytoplankton to Heterotrophic Bacterioplankton. Mbio.

[12] Chen J., Zhang J., Rosen B.P. (2019). Role of ArsEFG in Roxarsone and Nitarsone Detoxification and Resistance. Environ. Sci. Technol..

[13] Galván A.E., Paul N.P., Chen J., Yoshinaga-Sakurai K., Utturkar S.M., Rosen B.P., Yoshinaga M. (2021). Identification of the Biosynthetic Gene Cluster for the Organoarsenical Antibiotic Arsinothricin. Microbiol. Spectr..

[14] Chen J., Zhang J., Wu Y.F., Zhao F.J., Rosen B.P. (2021). ArsV and ArsW provide synergistic resistance to the antibiotic methylarsenite. Environ. Microbiol..

[15] Qin J., Rosen B.P., Zhang Y., Wang G., Franke S., Rensing C. (2006). Arsenic detoxification and evolution of trimethylarsine gas by a microbial arsenite S-adenosylmethionine methyltransferase. Proc. Natl. Acad. Sci. USA.

[16] Chauhan N.S., Ranjan R., Purohit H.J., Kalia V.C., Sharma R. (2009). Identification of genes conferring arsenic resistance to *Escherichia coli* from an effluent treatment plant sludge metagenomic library. FEMS Microbiol. Ecol..

[17] Chen J., Zhang J., Rosen B.P. (2022). Organoarsenical tolerance in *Sphingobacterium wenxiniae*, a bacterium isolated from activated sludge. Environ. Microbiol..

[18] Chen J., Bhattacharjee H., Rosen B.P. (2015). ArsH is an organoarsenical oxidase that confers resistance to trivalent forms of the herbicide monosodium methylarsenate and the poultry growth promoter roxarsone. Mol. Microbiol..

[19] Chen J., Yoshinaga M., Garbinski L.D., Rosen B.P. (2016). Synergistic interaction of glyceraldehydes-3-phosphate dehydrogenase and ArsJ, a novel organoarsenical efflux permease, confers arsenate resistance. Mol. Microbiol..

[20] Shi K., Li C., Rensing C., Dai X., Fan X., Wang G. (2018). Efflux Transporter ArsK Is Responsible for Bacterial Resistance to Arsenite, Antimonite, Trivalent Roxarsone, and Methylarsenite. Appl. Environ. Microbiol..

[21] Chen J., Madegowda M., Bhattacharjee H., Rosen B.P. (2015). ArsP: A methylarsenite efflux permease. Mol. Microbiol..

[22] Mukhopadhyay R., Rosen B.P. (2002). Arsenate reductases in prokaryotes and eukaryotes. Environ. Health Perspect..

[23] Lopez-Maury L., Sanchez-Riego A.M., Reyes J.C., Florencio F.J. (2009). The glutathione/glutaredoxin system is essential for arsenate reduction in *Synechocystis sp*. strain PCC 6803. J. Bacteriol..

[24] Wang L., Chen S., Xiao X., Huang X., You D., Zhou X., Deng Z. (2006). *arsRBOCT* arsenic resistance system encoded by linear plasmid pHZ227 in *Streptomyces sp*. strain FR-008. Appl. Environ. Microbiol..

[25] Kim E., Yang S.M., Kim D., Kim H.Y. (2022). Complete Genome Sequencing and Comparative Genomics of Three Potential Probiotic Strains, *Lacticaseibacillus casei* FBL6, *Lacticaseibacillus chiayiensis* FBL7, and *Lacticaseibacillus zeae* FBL8. Front. Microbiol..

[26] Su J. (2020). Isolation, Identification and Arsenic Resistant Mechanism Analysis of an Arsenic Tolerant Bacteria *Paenibacillus taichungensis* NC1 (In Chinese). Master Thesis.

[27] Moe B., Peng H., Lu X., Chen B., Chen L.W.L., Gabos S., Li X.F., Le X.C. (2016). Comparative cytotoxicity of fourteen trivalent and pentavalent arsenic species determined using real-time cell sensing. J. Environ. Sci..

[28] Wilson S.C., Lockwood P.V., Ashley P.M., Tighe M. (2010). The chemistry and behaviour of antimony in the soil environment with comparisons to arsenic: A critical review. Environ. Pollut..

[29] Paez-Espino A.D., Nikel P.I., Chavarria M., de Lorenzo V. (2020). ArsH protects *Pseudomonas putida* from oxidative damage caused by exposure to arsenic. Environ. Microbiol..

[30] Gu J., Yao J., Duran R., Sunahara G. (2020). Comprehensive genomic and proteomic profiling reveal *Acinetobacter johnsonii* JH7 responses to Sb(III) toxicity. Sci. Total Environ..

[31] Rong Q., Ling C., Lu D., Zhang C., Zhao H., Zhong K., Nong X., Qin X. (2022). Sb(III) resistance mechanism and oxidation characteristics of *Klebsiella aerogenes* X. Chemosphere.

[32] Maier J., Witter K., Gutlich M., Ziegler I., Werner T., Ninnemann H. (1995). Homology cloning of GTP-cyclohydrolase I from various unrelated eukaryotes by reverse-transcription polymerase chain reaction using a general set of degenerate primers. Biochem. Biophys. Res. Commun..

[33] Maita N., Hatakeyama K., Okada K., Hakoshima T. (2004). Structural basis of biopterin-induced inhibition of GTP cyclohydrolase I by GFRP, its feedback regulatory protein. J. Biol. Chem..

[34] Auerbach G., Herrmann A., Bracher A., Bader G., Gutlich M., Fischer M., Neukamm M., Garrido-Franco M., Richardson J., Nar H. (2000). Zinc plays a key role in human and bacterial GTP cyclohydrolase I. Proc. Natl. Acad. Sci. USA.

[35] Basset G., Quinlivan E.P., Gregory J.F., Hanson A.D. (2005). Folate synthesis and metabolism in plants and prospects for biofortification. Crop Sci..

[36] Kile M.L., Ronnenberg A.G. (2008). Can folate intake reduce arsenic toxicity?. Nutr. Rev..

[37] Bozack A.K., Hall M.N., Liu X., Ilievski V., Lomax-Luu A.M., Parvez F., Siddique A.B., Shahriar H., Uddin M.N., Islam T. (2019). Folic acid supplementation enhances arsenic methylation: Results from a folic acid and creatine supplementation randomized controlled trial in Bangladesh. Am. J. Clin. Nutr..

[38] Hossain T., Rosenberg I., Selhub J., Kishore G., Beachy R., Schubert K. (2004). Enhancement of folates in plants through metabolic engineering. Proc. Natl. Acad. Sci. USA.

[39] Sybesma W., Starrenburg M., Kleerebezem M., Mierau I., de Vos W.M., Hugenholtz J. (2003). Increased production of folate by metabolic engineering of *Lactococcus lactis*. Appl. Environ. Microbiol..

[40] Mansour N.M., Sawhney M., Tamang D.G., Vogl C., Saier M.H. (2007). The bile/arsenite/riboflavin transporter (BART) superfamily. FEBS J..

[41] Fu H.L., Meng Y., Ordonez E., Villadangos A.F., Bhattacharjee H., Gil J.A., Mateos L.M., Rosen B.P. (2009). Properties of arsenite efflux permeases (Acr3) from *Alkaliphilus metalliredigens* and *Corynebacterium glutamicum*. J. Biol. Chem..

[42] Wysocki R., Bobrowicz P., Ulaszewski S. (1997). The *Saccharomyces cerevisiae ACR3* gene encodes a putative membrane protein involved in arsenite transport. J. Biol. Chem..

[43] Garbinski L.D., Rosen B.P., Chen J. (2019). Pathways of arsenic uptake and efflux. Environ. Int..

[44] Lopéz Y.P., da Fonseca Breda F.A., Lima E.S.A., da Costa Barros de Souza C., González J.M.F., do Amaral Sobrinho N.M.B. (2021). Variability factors of heavy metals in soils and transfer to pasture plants of Mayabeque in Cuba. Environ. Monit. Assess..

[45] Yu J., Zhou X.F., Yang S.J., Liu W.H., Hu X.F. (2013). Design and application of specific 16S rDNA-targeted primers for assessing endophytic diversity in *Dendrobium officinale* using nested PCR-DGGE. Appl. Microbiol. Biotechnol..

[46] Kumar S., Nei M., Dudley J., Tamura K. (2008). MEGA: A biologist-centric software for evolutionary analysis of DNA and protein sequences. Brief. Bioinform..

[47] Yuan Z., Ruan J., Li Y., Qiu R. (2018). A new model for simulating microbial cyanide production and optimizing the medium parameters for recovering precious metals from waste printed circuit boards. J. Hazard. Mater..

[48] Wang K., Li Y., Wu Y., Qiu Z., Ding Z., Wang X., Chen W., Wang R., Fu F., Rensing C. (2020). Improved grain yield and lowered arsenic accumulation in rice plants by inoculation with arsenite-oxidizing *Achromobacter xylosoxidans* GD03. Ecotoxicol. Environ. Saf..

[49] Beringer J.E. (1974). R factor transfer in Rhizobium leguminosarum. J. Gen. Microbiol..

[50] Tatusova T., DiCuccio M., Badretdin A., Chetvernin V., Nawrocki E.P., Zaslavsky L., Lomsadze A., Pruitt K.D., Borodovsky M., Ostell J. (2016). NCBI prokaryotic genome annotation pipeline. Nucleic Acids Res..

[51] Carlin A., Shi W., Dey S., Rosen B.P. (1995). The *ars* operon of *Escherichia coli* confers arsenical and antimonial resistance. J. Bacteriol..

