# Supplementary material for: Characterization of Two Highly Arsenic-Resistant Caulobacteraceae Strains of Brevundimonas nasdae: Discovery of a New Arsenic Resistance Determinant
Source: Int J Mol Sci. 2022 May 17;23(10):5619. doi: 10.3390/ijms23105619 (PMC9144182; doi:10.3390/ijms23105619)
Supplement: Supplementary file 1 [file ijms-23-05619-s001.zip › ijms-1696850-supplementary.pdf]

Supplementary data

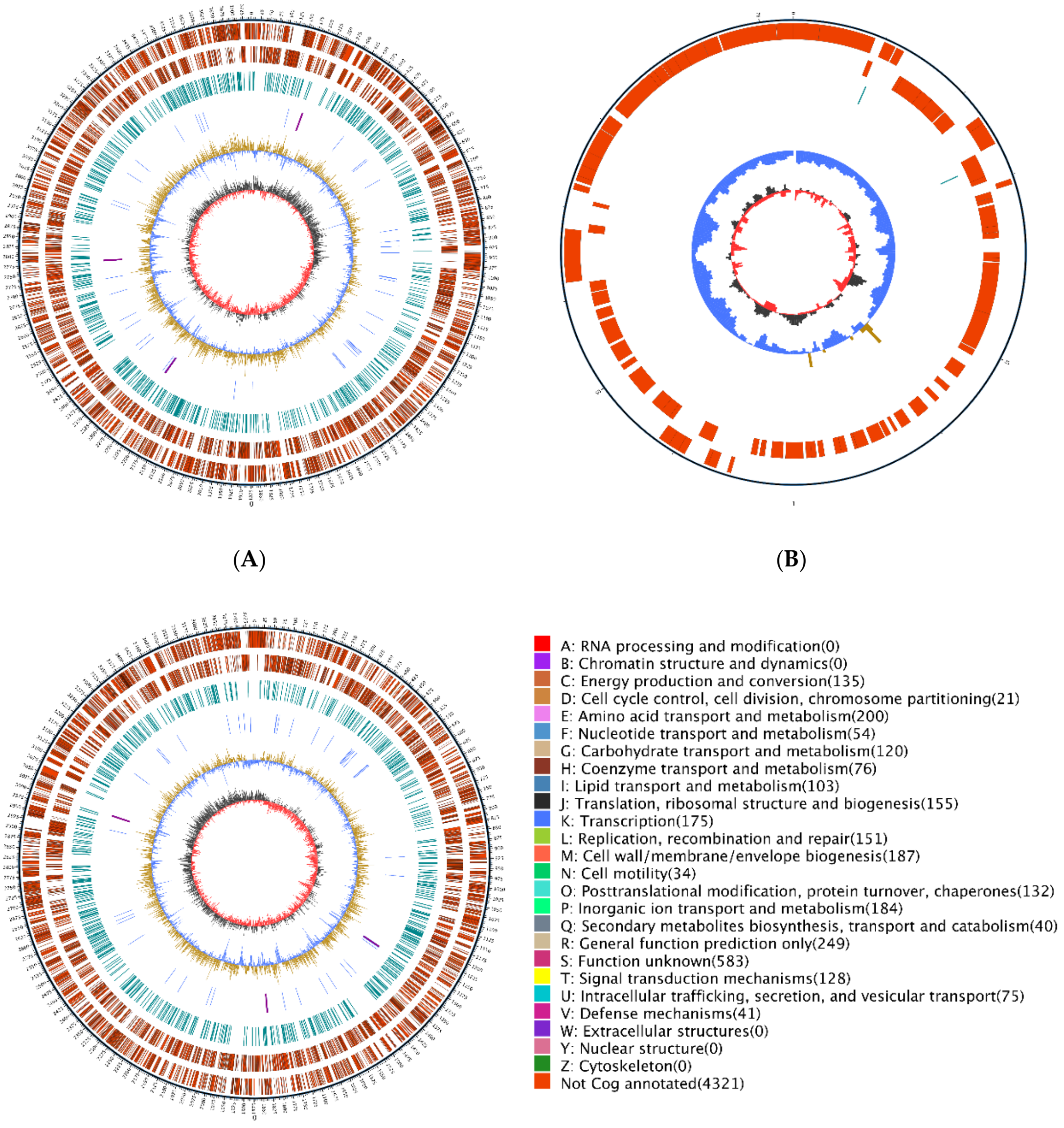

(**C**)

Figure S1. Circular map of the complete genome of *B. nasdae* Au-Bre29 and Au-Bre30. (**A**) Chromosome of Au-Bre29, (**B**) Plasmid of Au-Bre29 and (**C**) Chromosome of Au-Bre30. Circles from the outwards to inwards, ring 1: genome size (Kb); ring 2 and 3: genes existing on the Forward strand and Reverse strand, with different color indicate genes with different COG function; ring 4: repetitive sequence; ring 5: tRNA(blue) and rRNA(purple); ring 6: GC-content, with blue indicating the region with GC-content higher than average, light yellow indicate the region with GC-content lower than average; ring 7: GC-skew.

Table S1. General genomic feature of *B. nasdae* Au-Bre29 and Au-Bre30

Genomic contents	*B. nasdae* Au-Bre29	*B. nasdae* Au-Bre30
**Bio Project Number**	PRJNA744386	PRJNA744387
**NCBI Accession**	NZ_CP080034.1	NZ_CP080036.1
	NZ_CP080035.1	
**Chromosome**	1 (3.747438 Mb)	1 (3.746705 Mb)
**Plasmid**	1 (0.076784 Mb)	0
**G+C content (%)**	65.69%	65.8%
**Genome size (Mp)**	3.8251164	3.746705
**Total CDS**	3665	3577
**Total tRNAs**	53	53
**Total Genes**	3731	3643
**Protein coding genes**	3618	3531
**Genes with function prediction**	3531	3474
**Genes assigned to KEGGs**	1772	1751
**Genes with signal peptides**	531	523
**Genes assigned to VFDB (Virulence)**	670	668
**Clustered gene**	8	9
**Gene encoding transport protein**	888	882
**Gene encoding secreted protein**	531	523
**Genes assigned to CARD (Antibiotic)**	8	6
**Genes assigned to TCDB (Transport**	888	875

**protein)**		
**Genes assigned to CAZy (EPS)**	139	138
**CRISPR**	3	2
**GIs**	9	9
**Prophage**	5	4
**Phage**	3(1/3 intact)	3(1/3 intact)

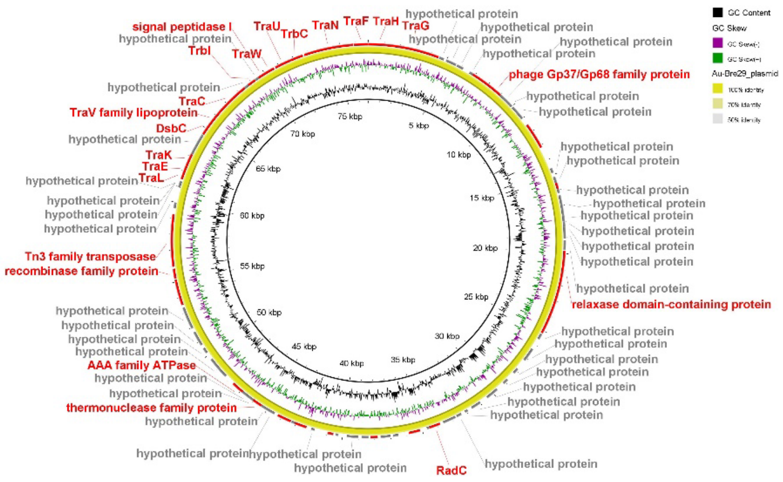

Figure S2. Plasmid circular map of *B. nasdae* Au-Bre29. Generated by BLAST Ring Image Generator (BRIG), shows the products of genes and their locations.

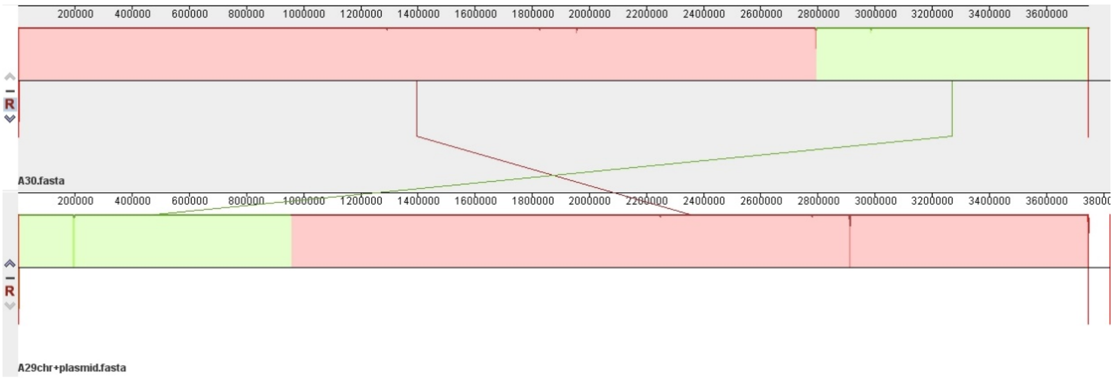

Figure S3. Comparison between the complete genomes of *B. nasdae* Au-Bre29 and Au-Bre30 by Mauve. Regard Au-Bre30 as reference genome (the top one).

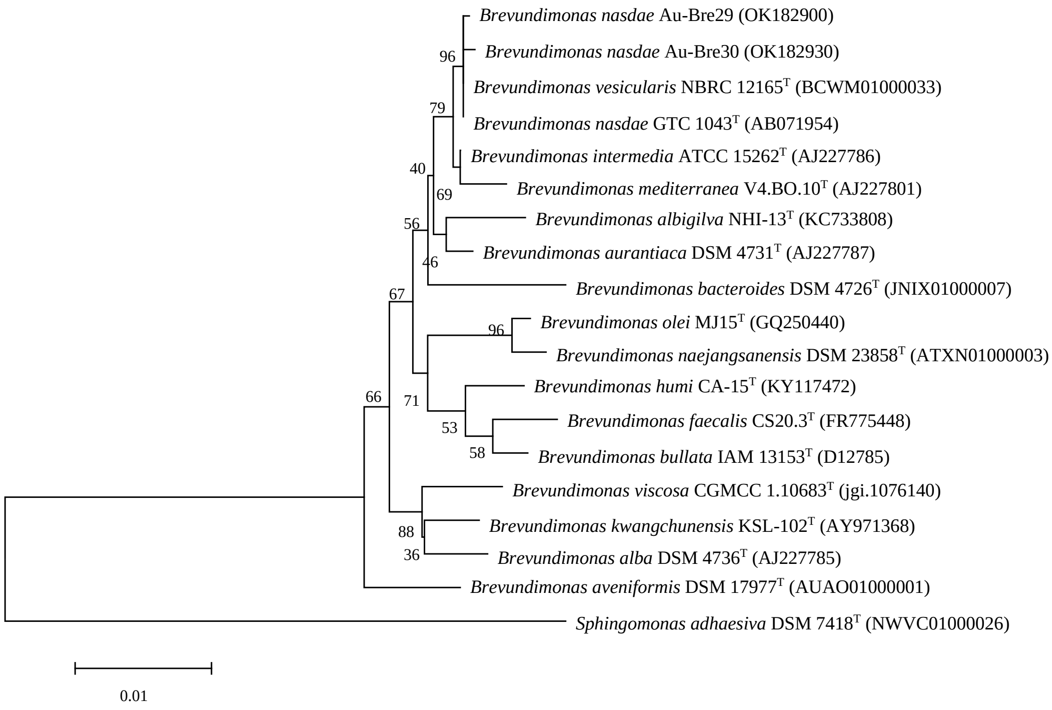

Figure S4. Neighbor-joining phylogenetic tree constructed based on the 16S rRNA gene sequences. Showing the phylogenetic relationships between strains *Brevundimonas nasdae* Au-Bre29 and Au-Bre30 and other species in the genus *Brevundimonas*. Values indicate percentages of identical branching in 1000 bootstrap replications. The sequence of *Sphingomonas adhaesiva* DSM 7418^T was used as an out-group. Bar, 0.01 substitutions per nucleotide position.
